# What will radiation oncology look like in 2050? A look at a changing professional landscape in Europe and beyond

**DOI:** 10.1002/1878-0261.12731

**Published:** 2020-06-30

**Authors:** Michael Baumann, Nadja Ebert, Ina Kurth, Carol Bacchus, Jens Overgaard

**Affiliations:** ^1^ German Cancer Research Center (DKFZ) Heidelberg Germany; ^2^ OncoRay—National Center for Radiation Research in Oncology Medical Faculty and University Hospital Carl Gustav Carus Technische Universität Dresden Germany; ^3^ Ruprecht‐Karls‐University Heidelberg Germany; ^4^ Department of Experimental Clinical Oncology Aarhus University Hospital Denmark

**Keywords:** anticancer strategies, early detection, health care, multidisciplinary treatment, personalized oncology, radiation oncology

## Abstract

The number of newly diagnosed cancers per year is predicted to almost double in the next two decades worldwide, and it remains unclear if and when this alarming trend will level off or even reverse. As such, cancer is very likely to continue to pose a major threat to human health. Radiation oncology is an indispensable pillar of cancer treatment and a well‐developed discipline. Nevertheless, key trends in cancer research and care, including improved primary prevention, early detection, integrated multidisciplinary approaches, personalized strategies at all levels of care, value‐based assessments of healthcare systems, and global health perspectives, will all shape the future of radiation oncology. Broader scientific advances, such as rapid progress in digitization, automation, and in our biological understanding of cancer, as well as the wider societal view of healthcare systems will also influence radiation oncology and how it is practiced. To stimulate a proactive discussion on how to adapt and reshape our discipline, this review provides some predictions on what the role and practice of radiation oncology might look like in 30 years’ time.

## Graphical abstract

This review aims to stimulate a proactive discussion on what radiation oncology might look like in 2050 and how the role and practice of radiation oncologists can be adjusted to reduce cancer incidence and improve cancer treatment. Rapid advances in digitization, automation, our biological understanding of cancer and our future healthcare systems will transform the field of radiation oncology.

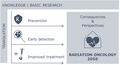

**Abbreviations**
IMRT: intensity‐modulated radiotherapy
MRI: magnetic resonance imaging
SBRT: stereotactic body radiotherapy
VMAT: volume modulated arc therapy
WHO: World Health Organization

## 1 Introduction

Today, cancer treatment is a multidisciplinary process that involves oncologists from different disciplines working together to ensure that each patient receives optimal treatment (Prabhu Das *et al.*, 2018; Selby *et al.*, 2019). Radiotherapy is a fundamental component of effective cancer therapy; currently, ~50% of all cancer patients require radiotherapy during the course of their disease (Atun *et al.*, 2015; Jaffray *et al.*, 2015). According to the World Health Organization (WHO), there were more than 250 new cancer cases in 2018, which resulted in more than 100 deaths per 100 000 inhabitants per year in Europe (http://gco.iarc.fr/today/home). It is forecasted that the incidence of newly diagnosed cancer cases worldwide will significantly increase from today’s 18.1 million to 29.5 million by 2040 (http://gco.iarc.fr/today/). This significant increase can be attributed to the growth of the world population in conjunction with an increased life expectancy in a number of countries, as well as to changes in lifestyle.

In Europe, however, the situation is different because it is the only continent in which the population is predicted to decrease in the years to come. At the same time, demographic developments in Europe are leading to a more marked increase in the elderly population at risk of developing cancer. Today, around 3.9 million new cancer patients are registered in Europe per year, of which 60% are over 65 years old (Eggermont *et al.*, 2019; Malvezzi *et al.*, 2019; Schuz *et al.*, 2019; Wild, 2019). In 20 years, this number will increase to 4.5 million, with 65% and 50% of patients being over 65 and 75 years of age, respectively. By 2050 therefore, the typical cancer patient in Europe will be 70 years or older. As such, the patients we currently consider to be elderly and more fragile, and who we therefore often exclude from clinical trials, will be the typical cancer patients of tomorrow (Overgaard, 2015). Concomitantly, thanks to better medical care, Europe will see a sharp increase in the number of citizens who are living with cancer or who have received successful cancer treatment (Lagergren *et al.*, 2019). These cancer patients will have a longer life expectancy. We therefore expect to see an increase in late treatment sequelae in conjunction with comorbidity and impaired quality of life.

**Fig. 1:**
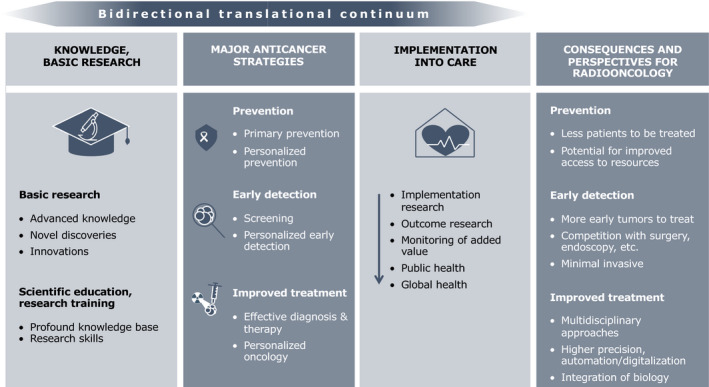
Strategies to decrease cancer incidence or death and their predicted impact for radiation oncology in 2050.

From these predicted numbers, it is obvious that cancer will pose an enormous and growing challenge to healthcare systems worldwide. To counteract these alarming trends, considerable investments into both cancer research and care are needed. We envision that scientific discoveries and innovations will feed into three major anticancer translational strategies: (a) primary prevention; (b) early detection; and (c) improved treatment (Zeggini *et al.*, 2020). All of these strategies will have a profound impact on the practice of radiation oncology in 2050 (Fig. 1).

## 2 Primary prevention

Likely the best, and probably also the only way to decrease the incidence of cancer and thus reduce the overall cancer burden from a medical and societal perspective is to prevent the development of cancer from the outset. Primary prevention through the avoidance of cancer‐associated risk factors, such as tobacco, physical inactivity, unhealthy dietary factors and levels of alcohol consumption, obesity, and overweightness, as well as avoiding exposure to direct or indirect infectious carcinogens, environmental pollutions, occupational carcinogens, and radiation, could together prevent 30–50% of all new cancer cases (https://www.who.int/news‐room/fact‐sheets/detail/cancer; Behrens *et al.*, 2018; Gredner *et al.*, 2018; Islami *et al.*, 2018; Mons *et al.*, 2018; Renehan *et al.*, 2008). The enormous potential of primary prevention to reduce the number of new cancer cases clearly calls for a strengthening of research into cancer prevention and associated public health strategies.

So how would improvements in primary prevention effect radiation oncology? One consequence would be a decline in the number of cancer patients who need to be treated. This is of great importance for the future provision of optimal radiation treatment for all cancer patients in need, which is currently neither the case on a global nor European level because the number of patients exceeds the available radiation oncology treatment resources (Atun *et al.*, 2015; Jaffray *et al.*, 2015). However, given that it often takes many years from the time of a cancer’s induction to it producing tumors that cause clinical symptoms, it will be many years before the positive effects of primary prevention will be felt by radiotherapy services. Nevertheless, radiation oncologists should be aware of the opportunities that arise from improved prevention, and they should support the efforts of researchers and public health professionals to raise public awareness and to help enforce national prevention programs and policies. Public health sciences and practical applications should also play a more prominent role in radiation oncology training. The inclusion of these disciplines in the curricula of radiation oncology training programs will not only support the long‐term impact on global health and prevention programs (in terms of improving patient access to radiotherapy) but will also help radiation oncologists to better utilize epidemiological data to plan their services and to cope appropriately with acute public health threats, such as the current COVID‐19 pandemic, which is having a significant impact on cancer care (Baumann *et al.*, 2020, van de Haar *et al.*, 2020).

## 3 Early detection of cancer

Clearly, not all cancers can be avoided by primary prevention even if all citizens were to strictly follow and participate in prevention programs. It is estimated that up to two‐thirds of all cancers are caused by as yet unrecognized risk factors, including hereditary factors, or by stochastic mutations (Tomasetti *et al.*, 2017). However, if tumors are diagnosed at the very early stages of the disease, they can usually be cured with methods that are widely available already today. Thus, the early detection of cancer (i.e., secondary prevention) is a very important strategy for improving the outcome of cancer care in the coming decades. The cancer field has already made promising progress in understanding the biology of cancer induction and progression, and has developed novel molecular and imaging techniques. In the future, we envisage citizens being provided with a detailed family history and genetic risk profile should they ask for one. Depending on the identified risk score and on their specific lifestyle factors, citizens will then undergo medical testing for the early detection of cancer, according to personalized schedules. Such a personalized testing program could include the use of innovative molecular assays based on, for example, liquid biopsies. If biomarkers for tumor growth are detected, possibly even indicating the organ of origin, imaging methods such as magnetic resonance imaging (MRI) or endoscopy could be used to localize the tumor for curative treatment (Alix‐Panabieres and Pantel, 2016; De Palma and Hanahan, 2012; Hanash *et al.*, 2011; Rodriguez‐Martin *et al.*, 2020). Overall, the improved early detection of cancer is expected to result in a higher proportion of curable cancers being diagnosed, thus increasing the clinical importance of single, local cancer treatment modalities, such as tumor removal or treatment by endoscopy, surgery, or radiotherapy. Theoretically at least, the improved early detection of tumor types, such as colon, breast, cervix, or lung, might also translate into a reduction in cancer death rates that is more rapid than that achieved by primary prevention programs.

For radiation oncologists, improvements in the early detection of cancer will have considerable consequences for their future practice. For example, ablative stereotactic radiotherapy (with photons or particles, see Box 1) and brachytherapy (see Box 1) might be developed for a wider spectrum of tumor sites than those that are currently treated. These radiotherapy techniques will compete with minimal invasive surgery or endoscopy approaches and would therefore need to show evidence of better patient outcomes or lower costs. Multidisciplinary services provided from single care centers in form of ‘one‐stop‐shop‐services’ might also grow in importance as the toxicities expected from the treatment of early stage tumors will be low. Overall, and even more than is the case today, treatment decisions concerning early stage tumors will be based on functional outcome, biological age, biomarkers, and health economics. Thus, radiation oncologists should intensify research programs on early stage tumors to generate the evidence needed for future interdisciplinary decision making.

Box 1 **Glossary of terms**

- Stereotactic ablative radiotherapy/stereotactic body radiotherapy (SABR/SBRT) is a very focused radiation treatment approach that delivers a high radiation dose concentrated on the tumor, with a sharp fall‐off of the dose to the surrounding normal tissue. Stereotactic radiotherapy is particular advantageous in small tumors.
- Brachytherapy is a form of internal radiation therapy in which a sealed radioactive source is placed inside or in the immediate vicinity of the tumor to be irradiated.
- Inter‐ and intrafraction motion refers to movement of the tumor relative to the surrounding normal tissue from one radiotherapy session to the next or in the latter case during one session. Underlying reasons include growth or shrinkage of the tumor, changes in bladder or bowel filling, or movement caused by breathing or heartbeat. Counteracting these reasons by motion control measures becomes more and more important with increasing precision of radiotherapy.
- FLASH radiotherapy is based on very high dose rates (dose rate 40–50 Gy·s^−1^ or more), which is believed to significantly reduce normal tissue damage without decreasing the effects of radiation on the tumor.
- Intensity‐modulated radiotherapy (IMRT) is a high precision way of irradiating the tumor where the intensity of the radiation dose can be changed within each irradiation field so that the delivered dose can be precisely adjusted point by point to the target volume.
- Particle and proton irradiation is a precision method of radiation therapy in which the tumor is irradiated with high‐energy positive ions (usually protons or carbon ions). Because of their inverse depth dose profile with a Bragg peak, dose to normal tissues can often be reduced compared to irradiation with photon beams. The relative biological effectiveness of particle beams is different to photon beams, which needs to be considered in treatment planning.
- Volume modulated arc therapy (VMAT) is a further development of IMRT that delivers the intensity‐modulated radiation dose continuously as the gantry of the treatment machine rotates over defined angular ranges shaping the radiation dose to the tumor.
- Radiobiology as a field of medical and clinical science studies the biological effects of ionizing radiation, for example, photons or carbon ions, on living organisms.

## 4 Improved cancer treatment

Even with improved early detection, a significant proportion of cancers will still only be detected at locally advanced or metastatic stages. The treatment of advanced cancer has been the mainstay of radiation oncology in most countries during recent decades, and enormous advances have been made in this field (Baumann *et al.*, 2016). Such advances include the development of technologies that increase conformality of the prescribed dose to the tumor while sparing normal tissue, such as intensity‐modulated radiotherapy (IMRT), volume modulated arc therapy (VMAT), stereotactic body radiotherapy (SBRT), and particle therapy (see Box 1). Image guidance is used in all of these techniques, and inter‐ and intrafraction motion control (see Box 1) have increasingly become a clinical reality. New hybrid radiotherapy devices that allow precise tumor imaging during radiation, that is, the recently introduced MR‐Linac system, further support clinical radiation oncology. Clinical and biological sciences have also advanced our field. For example, advances in these fields have supported the introduction of combined modality treatments for a wide range of tumors, the exploration of fractionation schedules and dose–volume relationships, and the development of radiotherapy from a stand‐alone discipline to an essential and well‐integrated area of expertise within multidisciplinary and multiprofessional cancer teams. A guideline‐driven, multidisciplinary approach is of fundamental importance for securing optimal treatment for all patients, in which the use of radiotherapy is balanced with treatment options provided by other disciplines. Ongoing clinical trials are key in this regard, as they provide the basis for the development of optimal treatment guidelines that support clinical decision making. As our clinical strategies evolve and change, some indications might disappear, while other new indications arise. For example, there is increasing evidence that the use of radiotherapy to treat local or metastatic disease in patients with a disseminated cancer might improve their outcome and, as in the case of oligo‐metastatic cancer, might even have curative potential (Guckenberger *et al.*, 2020).

## 5 Scientific discoveries and innovation

In the coming years, we expect to see a rapidly growing role for data science in cancer care and in medicine more generally, including the use of artificial intelligence and of big data analytics (Ngiam and Khor, 2019; Niazi *et al.*, 2019). No other cancer modality depends as much on modern IT technology as radiotherapy, and the field has advanced rapidly with increases in computational power. As a consequence, professionals in the field of radiation oncology are already significantly exposed to data science; for example, some already regularly use *in silico* model‐based approaches to produce personalized treatment plans for patients. As such, many radiation oncologists are well positioned to integrate data science further into their clinical practice, for example, by using prognostic and predictive biomarkers or by performing quantitative assessments of patient responses after they have received different multidisciplinary treatments. We believe that the time has come for radiation oncologists to team up with data scientists from different fields, for example, from medical informatics, bioinformatics, image analytics, biostatistics, and artificial intelligence, in order to harness the huge potential of data science for radiation oncology and multidisciplinary cancer care.

Another advance has come from the increased use of particle and proton irradiation (see Box 1) because of the higher precision of these techniques and because of other radiobiological considerations (Dutz *et al.*, 2019a; Dutz *et al.*, 2019b; Lühr *et al.*, 2018). Numerous new facilities that offer these treatment techniques are being established in Europe, and the potential clinical benefits they offer are being studied in a series of collaborative clinical trials, the outcomes of which are likely to define how such treatment will be used in 30 years’ time (Grau *et al.*, 2018; Grau *et al.*, 2020; Huynh *et al.*, 2019). But already, we can foresee that radiotherapy resources are likely to offer ‘a little to a lot and a lot to a little’. In such a scenario, most patients requiring radiotherapy would receive fairly standard and widespread radiotherapy techniques, in which most of the procedures are performed automatically, involving relatively little manpower. By contrast, other patients, including children, who have complex tumor locations or unfavorable outcomes following standard radiotherapy would receive, for example, particle therapy or another technologically advanced treatment. An undocumented guess, based on the expected outcome of the ongoing clinical trials, is that 10–15% of patients would qualify for the more technologically advanced types of treatments.

New variations in radiotherapy are also likely to be explored in the future. For example, there is currently significant interest in FLASH treatments (see Box 1), which entails physical and biological modifications to existing ionizing irradiation treatments (Bourhis *et al.*, 2019; Henry *et al*., 2017; Vozenin *et al.*, 2019). It is too early to say whether these modifications will have implications for future clinical strategies. However, new concepts and ideas that require scientific evaluation will continue to emerge along the way. One important factor to consider is that the radiobiological questions that we address often remain unchanged despite the technological improvements in treatment delivery and imaging (Overgaard, 2007). For example, hypoxia has been a challenge to radiotherapy for over 100 years, and we are no closer today to overcoming this biological hurdle.

While radiotherapy has always been a highly personalized cancer treatment regimen, with regard to clinical parameters and anatomic dose distribution, biology‐driven personalized radiotherapy enabling treatment based on the biological characteristics of the tumor and normal tissue is currently a promising research area in preclinical and clinical radiation oncology, and is finding its way into clinical practice (Baumann *et al.*, 2016; Linge *et al.*, 2016a; Linge *et al.*, 2016b; Lohaus *et al.*, 2014). In the future, this emerging field of radiobiology (see Box 1) will continue to team up with other fields of biological science and will be used by physicians and physicists in translational programs.

Another major issue for the field of radiotherapy is the rapid development and use of novel clinical trial designs for the era of personalized oncology. With increasing stratification of patients by biological parameters, the patient cohorts that can be enrolled into a given clinical trial at a single center are becoming smaller and smaller (Baumann *et al.*, 2016). This trend of molecular biological personalization is likely to continue as studies performed so far indicate important heterogeneity in radiation response between tumors of the same histology and stage in different patients (Baumann *et al.*, 2016; Linge *et al.*, 2016a; Linge *et al.*, 2016b; Lohaus *et al.*, 2014). This might lead to situations in which only a few patients can be recruited into trials, even at larger centers. In addition, trials are rapidly becoming increasingly complex, by requiring, for example, standardized molecular profiling, bioimaging, and repeat biopsies. This trend implies that large innovative networks and robust translational research platforms will need to be established to allow relevant trials to be pursued. Obviously, such networks and platforms would also need to cover radiation oncology specific research (Baumann *et al.*, 2016).

New biology‐driven challenges and opportunities for radiation oncologists will also arise from the dynamic translational developments that come from other fields of oncology. The recent introduction of immunotherapy as the fourth pillar of cancer treatment is a good example. Currently, it is still under debate whether the use of immune checkpoint inhibitors combined with radiotherapy will become a game‐changer in cancer treatment (Lambin *et al.*, 2019). Already numerous other immunotherapies, many of them cell‐based, are under development and testing. The use of these therapies will require radiobiology expertise if they are to be combined with radiation in the clinic (Mondini *et al.*, 2020).

Even when using the most advanced radiation technologies, the normal tissues of a patient are at risk of longer term effects of ionizing radiation. These harmful effects are dose‐, fractionation‐, volume‐, and organ‐dependent, and should be avoided as much as possible. Not only can they be modified by other therapies and by pre‐existing diseases, but they can also be associated with long observation periods and growth, making children particularly vulnerable patients (Lambrecht *et al.*, 2018). Elderly patients might also be at increased risk of such late‐onset effects since many of these effects add to age‐dependent reduction of the reserve capacity of a number of organs at risk (Bentzen, 2006). Due to an increasing prevalence of cured cancer patients, this has become a matter of concern that should be given a priority in future research.

In summary, these examples illustrate how scientific education and research are of key importance for the future professional role and development of radiation oncologists. They also support the view that academic radiation oncology departments must professionalize and expand their research laboratories, data science capabilities, and scientific staff in order to contribute efficiently to the growing demands of translational and clinical research, which are both indispensable for personalized cancer care.

## 6 Future perspective: What will radiation oncologists do in 2050?

### 6.1 Technology and research

Radiotherapy will become more closely integrated with different fields of diagnostic imaging because of the increasing importance of (biological) image guidance. There will be substantially more automated and robotic treatment options with fewer radio‐oncology activities, such as delineation, treatment planning, and supervision of radiotherapy delivery, performed by physicians and physicists. Radiation oncology research will thus be driven by a multiprofessional team consisting of (radio)biologists, physicists, engineers, data scientists, and clinical researchers.

### 6.2 Care

All cancer diagnostics and care will be carried out in a multidisciplinary setting, and specialized cancer teams will focus on specific cancer sites. Personalized oncology, including personalized radiation oncology, will become routine. Clinical decisions will be based on integrated molecular, imaging, clinical, and technological data. For this purpose, data science, including artificial intelligence and decision‐support systems, will be used, and the role of medical physicists and data scientists will expand. One‐stop‐shop approaches will gain in importance as cancer will be more often detected at an early stage. Radiotherapy will compete with alternative treatment options, such as minimal invasive surgery and other local therapies. The treatment of advanced disease will be multimodal, personalized, and both curative and palliative.

### 6.3 Training and education

Improved scientific education and knowledge in all areas of oncology will be needed to provide a more comprehensive picture of cancer as a disease and of anticancer treatment options. Radiation oncologists will be trained in public and global health approaches. The radiation oncologists’ role will shift toward science and to multidisciplinary decision making and to dedicated and intensive counseling during the patient’s treatment. Radiation therapy technologists and nurses will be academic professionals with seamless vertical career opportunities and with roles that overlap with today’s roles of radiation oncologist and medical physicist.

### 6.4 Society

Greater research will be performed into the effects of radiotherapy on elderly patients since a significantly larger proportion of patients will be elderly, many with comorbidities, who will need both gentle and effective treatment. Most cancer patients will live in low‐ or medium‐income countries. Global health efforts will have to be significantly strengthened to ensure that all patients can access radiotherapy when needed.

## 7 Conclusions

First of all, we firmly believe that radiation oncology will be at least as important in 2050 as it is today. A ‘magic bullet’ for cancer, as proposed by Nobel Laureate Paul Ehrlich in the early 20th century, is not only not in sight, but more and more unlikely in light of our increasing biological knowledge of this hugely heterogeneous disease (Consortium, 2020; De Palma and Hanahan, 2012). Radiotherapy has an impressive track record that demonstrates its curative potential in a wide variety of cancers. Given its unique features, radiotherapy will very likely remain a key component in the multidisciplinary, anticancer treatment arsenal of the future.

However, based on the above discussion, we anticipate that fundamental differences will occur in the daily practice of radiation oncology professionals in 2050, relative to their roles today. The year 2050 sounds far away and of little immediate concern. We disagree. Thirty years is less than a single generation’s turnover of health professionals, and today’s trainees will be the leaders of 2050. It also seems prudent to proactively design the future of radiation oncology now rather than to wait and merely react to what has happened around us. Educating the future leaders in radiation oncology—and not least ourselves—in a broader manner than in the past is of key importance for our field. Clearly, our predictions for the field must be taken with caution and adjusted overtime. As Niels Bohr once said: ‘Prediction is very difficult, especially if it’s about the future’. Nevertheless, the cancer and science trends we have outlined above provide a useful framework for further discussion.

## Conflict of interest

In the past 5 years, Dr. Michael Baumann attended an advisory board meeting of MERCK KGaA (Darmstadt), for which the University of Dresden received a travel grant. He further received funding for his research projects and for educational grants to the University of Dresden from Teutopharma GmbH (2011–2015), IBA (2016), Bayer AG (2016–2018), Merck KGaA (2014‐open), and Medipan GmbH (2014–2018). He is on the supervisory board of HI‐STEM gGmbH (Heidelberg) for the German Cancer Research Center (DKFZ, Heidelberg) and is also a member of the supervisory body of the Charité University Hospital, Berlin. As the former chair of OncoRay (Dresden) and as the current CEO and Scientific Chair of the German Cancer Research Center (DKFZ, Heidelberg), he has been or is responsible for collaborations with a numerous companies and institutions, worldwide. In this capacity, he discussed potential projects with and has signed/signs contracts for his institute(s) and for the staff for research funding and/or collaborations with industry and academia, worldwide, including but not limited to pharmaceutical corporations such as Bayer, Boehringer Ingelheim, Bosch, Roche, and other corporations like Siemens, IBA, Varian, Elekta, Bruker, and others. In this role, he has been and is responsible for commercial technology transfer activities of his institute(s), including the DKFZ‐PSMA617‐related patent portfolio [WO2015055318 (A1), ANTIGEN (PSMA)] and similar IP portfolios. Dr. Baumann confirms that to the best of his knowledge none of the above funding sources was involved in the preparation of this paper.

## Author contributions

All authors contributed to the writing and/or revision of this review article.
